# A silkworm based silk gland bioreactor for high-efficiency production of recombinant human lactoferrin with antibacterial and anti-inflammatory activities

**DOI:** 10.1186/s13036-019-0186-z

**Published:** 2019-07-05

**Authors:** Sheng Xu, Feng Wang, Yuancheng Wang, Riyuan Wang, Kai Hou, Chi Tian, Yanting Ji, Qianqian Yang, Ping Zhao, Qingyou Xia

**Affiliations:** 1 Biological Science Research Center, Southwest University, Chongqing, 400716 China; 2 Chongqing Key Laboratory of Sericultural Science, Southwest University, Chongqing, 400716 China; 3 Chongqing Engineering and Technology Research Center for Novel Silk Materials, Southwest University, Chongqing, 400716 China

**Keywords:** Recombinant human lactoferrin, High-efficiency production, Silkworm, Glycosylation modification

## Abstract

**Background:**

Silk glands are used by silkworms to spin silk fibers for making their cocoons. These have recently been regarded as bioreactor hosts for the cost-effective production of other valuable exogenous proteins and have drawn wide attention.

**Results:**

In this study, we established a transgenic silkworm strain which synthesizes the recombinant human lactoferrin (rhLF) in the silk gland and spins them into the cocoon by our previously constructed silk gland based bioreactor system. The yield of the rhLF with the highest expression level was estimated to be 12.07 mg/g cocoon shell weight produced by the transgenic silkworm strain 34. Utilizing a simple purification protocol, 9.24 mg of the rhLF with recovery of 76.55% and purity of 95.45% on average could be purified from 1 g of the cocoons. The purified rhLF was detected with a secondary structure similar with the commercially purchased human lactoferrin. Eight types of N-glycans which dominated by the GlcNAc (4) Man (3) (61.15%) and the GlcNAc (3) Man (3) (17.98%) were identified at the three typical N-glycosylation sites of the rhLF. Biological activities assays showed the significant evidence that the purified rhLF could relief the lipopolysaccharide (LPS)-induced cell inflammation in RAW264.7 cells and exhibit potent antibacterial bioactivities against the *Escherichia coli* (*E. coli*) and *Bacillus subtilis.*

**Conclusions:**

These results show that the middle silk gland of silkworm can be an efficient bioreactor for the mass production of rhLF and the potential application in anti-inflammation and antibacterial.

**Electronic supplementary material:**

The online version of this article (10.1186/s13036-019-0186-z) contains supplementary material, which is available to authorized users.

## Background

Human lactoferrin is an iron-binding glycoprotein [1] that is widely found in exocrine secretions including, milk, tears, saliva, bile, serum, gastrointestinal fluids, and vaginal fluids with high abundance [2]. Human lactoferrin has various biological functions, in particular, widely known bactericidal and bacteriostatic activities that form the primary line of defense against the microbial infections [3, 4]. For gram-positive bacteria (G^+^), the N-terminal basic amino acids of lactoferrin can combine with the lipoteichoic acid wall of the bacterial cell membrane, neutralising the wall charge and allowing the action of other antibacterial compounds such as lysozyme, and the bacteria lysis. For gram-negative bacteria (G^−^), the amino acid residues G^1^RRRRS^6^ [5] and R^28^KVRGPP^34^ [6] in the N-terminal domain of lactoferrin can bind specifically and with a high affinity to the lipid A regions of the bacterial LPS [7], which prompts the fast release of LPS from bacteria, increases the bacterial membrane permeability, destroys the lipid bilayer, and finally kill the bacteria. Meanwhile, the bacteriostatic property of lactoferrin is due to the capability of each lobe to bind Fe^3+^ and thus suppress bacterial growth by producing an iron-deficient environment [8]. Thus, diet supplementation on with lactoferrin is an effective strategy to reduce the occurrence of late onset sepsis in neonates and to control the development of acute septic inflammation in neonates [9]. In addition, human lactoferrin has many other biological functions including antitumor [10], antioxidant [11] and stimulate the proliferation in many cell types [12]. Therefore, human lactoferrin is regarded as a potential compound for the food, cosmetics, and feed additives applications.

Considering the potential therapeutic value and the global demand for human lactoferrin, it is of profound significance to seek a highly efficient system for the expression of human lactoferrin. For decades, synthesis of human lactoferrin has been attempted in various host systems such as the bacteria (*Lactobacillus casei*) [13], yeast (*Pichiapastoris*) [14], fungi (*Aspergillusawamori*) [15], NPV-infected insects (*Bombyxmori*) [16], cell lines (Baby hamster kidney) [17], mammals (goat, mice, rabbit, bovine) [18, 19], and plants (*Nicotiana tabacum*, rice, potato, sweet potato) [20–22]. However, poor yields of recombinant protein, lack of post-translational modification, and a complex expression process has severely hindered these expression system applications.

Silkworm is an economically important insect with a short life cycle, low feeding cost, protein post-translation modification, and possibility of easy scale-up. After more than 5000 years of domestication, silkworms are capable of synthesizing abundant silk proteins within a short term in their silk glands and spinning silk fibers to make their cocoons. Thus, mass production of recombinant proteins in the silk glands of silkworms has been in consideration for a long time. Silk proteins are majorly comprised the fibroin proteins and sericin proteins. Fibroin proteins contain fibroin heavy chains (H-chains), fibroin light chains (L-chains), and fibrohexamerins, and are synthesized by the posterior silk glands cells [23]. The sericin proteins are mainly encoded by sericin-1, sericin-2, and sericin-3 genes, and are specifically synthesized by the middle silk glands cells [24]. With the development of genetic manipulation tools, in particular the successful establishment of the *piggyBac* transposon-mediated genetic transformation technology in silkworms [25], it is possible to genetically engineer the silkworm silk gland as an ideal bioreactor for the recombinant expression of more valuable foreign proteins along with synthesis of their silk proteins [26]. Therefore, two major expression systems associated with the usage of promoters from the genes for fibroins and sericins have been constructed [27–29], and more than ten recombinant proteins with various bio-functions and application potential have been successfully expressed in the silk glands of transgenic silkworms and cocoons, including human collagen, human serum albumin, antibodies, and human growth factors [30–33]. These efforts have greatly booted the process of massive production of recombinant pharmaceutical proteins by the silk gland of silkworm to meet the daily increased clinical demands.

Considering the huge potential application value of human lactoferrin and the advantage of recombinant expression of exogenous proteins in the silk gland of the silkworm, we aimed to use the previously generated *sericin-1* expression system [29] to express rhLF. The transgenic silkworm integrated with an rhLF expression cassette was generated and the expression, purification, secondary structure, glycosylation modification, and in vitro anti-inflammation and antibacterial bioactivities of rhLF in the cocoon were studied and analyzed. The results strongly showed the feasibility of cost-effective and bulk production of rhLF with natural bioactivity using silk gland-based bioreactors of silkworm.

## Results

### Generation of transgenic silkworm

**Fig. 1:**
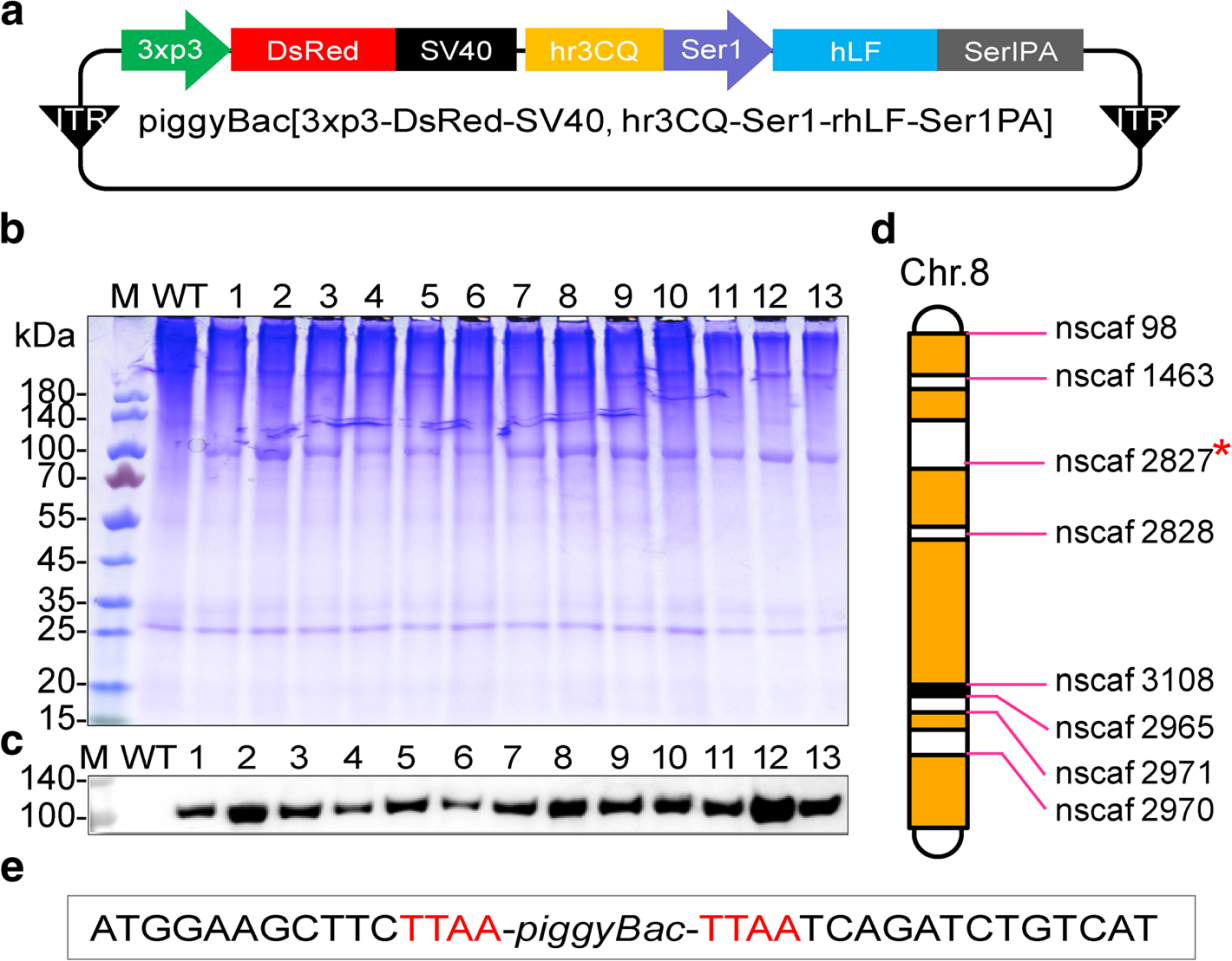
Generation of rhLF transgenic silkworm. **a** Structural map of the transgenic vector. 3xp3RFP represents the selection marker of the transgene; SV40 indicates the termination codon sequence of sericin1 gene; Ser1 represents the promoter region of the sericin1 gene; Ser1PA indicates the poly A sequence of the sericin1 gene. **b** SDS-PAGE analysis of the cocoon proteins from 13 different transgenic lines. Lane 1–13, 13 transgenic cocoon sericin extracts; M and WT represent the marker and wild type samples, respectively. **c** Western blot analysis of rhLF in sericin crude extract from number 1–13, the thicker strip indicates the higher rhLF content. The individual with the highest rhLF content was selected to established the rhLF transgenic line. **d** The insertion site of the rhLF gene on the chromosome of the transgenic silkworm. The highest expression level of the rhLF transgenic silkworm was determined, and then backcross with a normal moth, its offspring were used for insertion site detection after the establishment of the silkworm strain. **e** The insertion site of the transgene in the rhLF transgenic silkworm genome, the target sequence “TTAA” specifically recognized by *piggyBac* transposase is highlighted in red

To achieve efficient expression of rhLF in the middle silk gland of transgenic silkworm, a *piggyBac*-based transgenic vector phSrhLFSer1 was constructed (Fig. 1a). The coding sequence of the full length of rhLF with a 6 × His-tag at the C-terminal was artificially designed and synthesized according to the silkworm codon usage bias, and the expression of designed rhLF was spatially and temporally regulated by an hr3CQ enhancer activated sericin 1 promoter. The 3xp3-DsRed marker that achieves the specific expression of DsRed in the ocelli and compound eyes of silkworm [34, 35], was used for screening positive egg individuals. Then, 200 non-diapause silkworm embryos were microinjected with a mixture of plasmid consisting the phSrhLFSer1 and helper vector, among which, 42.0% injected embryos were successfully hatched and were carefully fed until the moth stage to oviposit the G1 offspring. In total, 43 G1 broods were obtained and among them, 12 (27.9%) broods were detected with RFP-positive individuals having specific DsRed emission in the ocelli of the body pigmentation stage in the embryos and the compound eyes of the moths (Additional file 1: Figure S1; Table 1), indicating the successful and efficient transformation of hereditable transgenesis in silkworms. Thereafter, all positive individuals from different silkworm broods were carefully reared and total 65 positive larva progressed to spin cocoons, which were then subjected to cocoon protein analysis by SDS-PAGE and western blot. The results showed a significantly visible protein band on the CBB-stained gel among the cocoon protein samples from all positive individuals compared to that from the wild-type (Fig. 1b). The molecular weight of these distinct protein bands was between 70 to 100 kDa, which is in accordance with the theoretical size of the rhLF (~ 80 kDa). Further immunoblot blot analysis showed that these distinct protein bands could react with the anti-human lactoferrin antibody (Fig. 1c). These results suggest that the rhLF was successfully expressed in the silk gland cells of transgenic silkworms and was secreted into the cocoons. Further band scan analysis showed that the content of the rhLF in the total extracted cocoon proteins from the 65 positive individuals was 8.53–14.77%, (Additional file 2: Figure S2) and the positive transgenic silkworm strain 34 with the highest expression of rhLF was maintained to generate a stable transgenic strain. Only a single band after PCR amplification using pBacL and pBacR primers suggesting that rhLF was inserted as a single copy (Additional file 4: Figure S4). Insertion of the rhLF expression cassette was mediated by the *piggyBac* transposon into a “TTAA” preference site [36] on Chr. 8, nscaf 2827: 8517795–8,518,010 of the silkworm genome (Fig. 1d-e).

**Table 1:** Results of transgenic vector microinjection

Vector	Injectable line	Injected embryos (G0)	Hatched	G1 broods	G1 positive broods	Positive rate of G1 generation (%)
PhSrhLFSer1	D9L	200	84	43	12	27.9%

### Purification and secondary structure determination of the rhLF

**Fig. 2:**
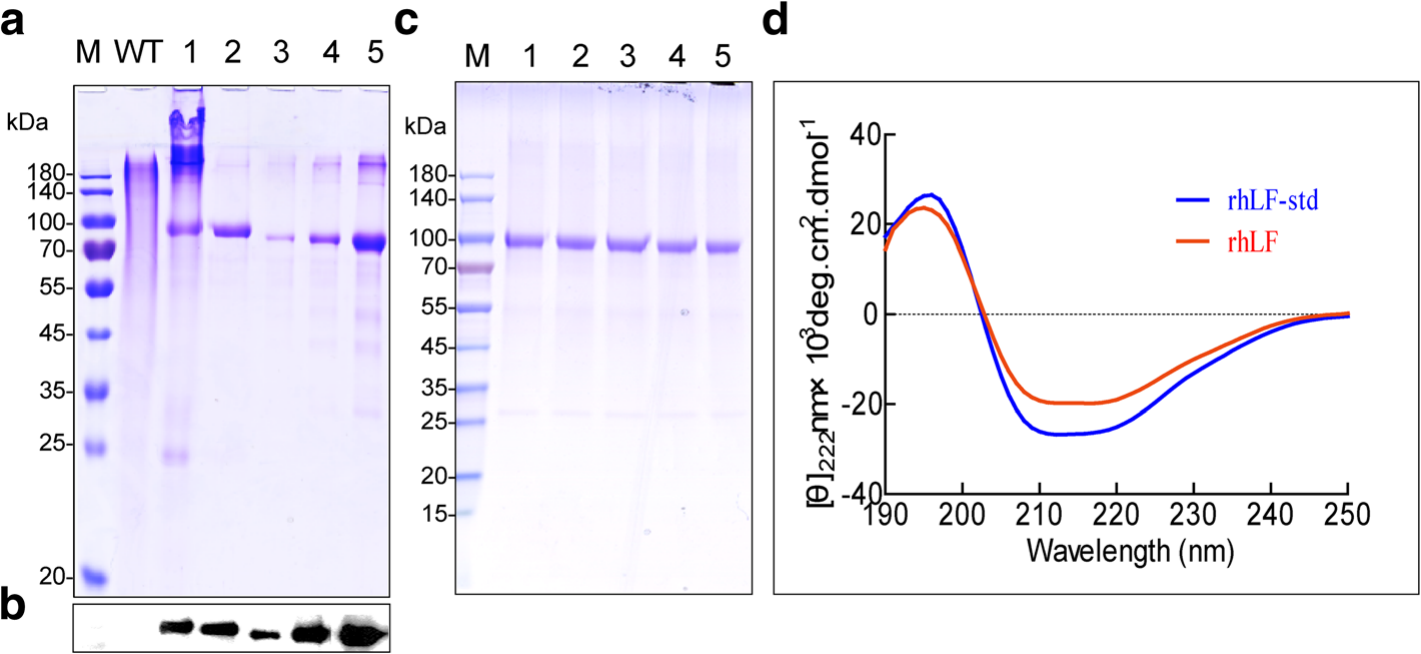
Purification and secondary structure analysis of rhLF. **a** Purification of rhLF. M and WT represent the marker and wild type samples, respectively. Lane 1 represents the crude extract from cocoons. Lane 2 represents purified rhLF. Lane 3–5 represent 100, 250, 500 μg/ml of the rhLF-std, respectively. **b** Western blot analysis of the rhLF. **c** Detection rhLF after purification and dialysis. M represents the marker. Lanes 1–5 represent 5 batches of purified and dialyzed rhLF. **d** CD spectra of rhLF and rhLF-std. The rhLF and rhLF-std at the same concentration (0.5 mg/ml) were scanned within 190–250 nm

A one-step immobilized metal-chelated affinity chromatography (IMAC) purification process using a Ni-charged His-binding column was designed to purify the rhLF from the cocoons. After a complete extraction, a total 12.07 mg rhLF could be recovered from 1 g cocoons from the transgenic silkworm No. 34 strain. The crude extract from the cocoons of transgenic silkworm No. 34 strain was flew through the Ni-charged His-binding column, washed with wash buffer containing 20 mM imidazole and was eluted with an elution buffer containing 35, 50, 100, 150, and 200 mM imidazole (Additional file 3: Figure S3). SDS-PAGE and western blotting showed that the rhLF recombinant protein was effectively purified from endogenous silk proteins (Fig. 2a-b). The purity of the obtained rhLF was estimated to be 95.17% by densitometry calculation and comparison with the rhLF standard (rhLF-std). Finally, 9.24 mg rhLF with a recovery of 76.55% could be purified from 1 g of cocoons from the transgenic silkworm No. 34 strain. Furthermore, the purity of rhLF from five independent processing steps was 95.45% with a variability coefficient of 0.82% (Fig. 2c), suggesting that the processing for rhLF purification is robust and highly reproducible. These results show that the silk gland offers a cost-effective alternative for the rhLF production. The secondary structure of the purified rhLF was then determined by Far-UV circular dichroism (CD) spectra. The result showed that at the same concentration, rhLF and the rhLF-std have a similar CD map between 190 and 250 nm, two typical negative bands at 208 nm and 222 nm, and a positive band at 193 nm on the map (Fig. 2d). Further calculation and analysis using CD Pro showed that the two samples contain considerablely similar *α*-helix, *β-*fold, *β-*angle, and irregular curl structures (Table 2). These results indicate that the rhLF expressed in the MSG of the silkworm has a similar secondary structure as that of the commercial rhLF-std.

**Table 2:** Contents of the secondary structure of rhLF and rhLF-std

Samples	*α*-helix (%)	*β-*fold (%)	*β-*angle (%)	irregular curl (%)
rhLF-std	52.60 ± 1.30	10.35 ± 0.85	14.90 ± 2.20	22.50 ± 0.60
rhLF	51.20 ± 2.15	10.90 ± 1.20	15.30 ± 1.50	22.90 ± 1.10

### Identification of N-glycosylation of rhLF

**Fig. 3:**
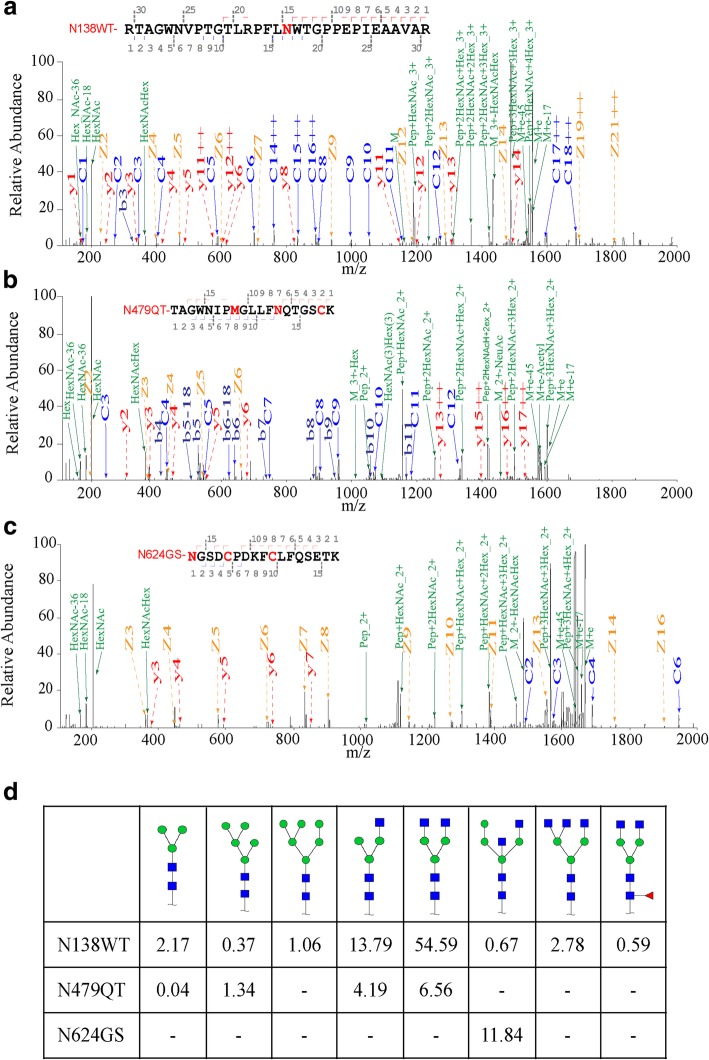
N-glycosylation analysis of rhLF. **a** MS/MS spectra of N-glycopeptide RTAGWNVPTGTLRPFLNWTGPPEPIEAAVAR (AA 122–152) of rhLF. **b** MS/MS spectra of N-glycopeptide TAGWNIPMGLLFNQTGSCK (AA 467–485) of rhLF. **c** MS/MS spectra of N-glycopeptide NGSDCPDKFCLFQSETK (AA 627–641). **d** Percentage distributions of glycoforms were calculated by relative peak area average values. Green circle represent mannose; blue square represents N-acetylglucosamine; red triangle represents fucose.

Peptide mapping liquid chromatography/mass spectrometry (LC/MS) was performed to reveal the N-glycosylation of rhLF produced in the MSG of transgenic silkworm. The LC/MS data of the rhLF showed a 96.18% match with human lactoferrin amino acid reference (Additional file 5: Figure S5). Three typical N-glycosylation sites of human lactoferrin, Asn-138, Asn-479, and Asn-624 were detected (Fig. 3a-c). Eight types of N-glycan fractions were obtained, and their structures were analyzed and identified as GlcNAc (2) Man (3), GlcNAc (2) Man (5), GlcNAc (2) Man (6), GlcNAc (3) Man (3), GlcNAc (4) Man (3), GlcNAc (4) Man (4), GlcNAc (5) Man (3), and GlcNAc (4) Man (3) Fuc (1). The quantitative analysis showed that the major N-glycans were of hybrid-type such as GlcNAc (3) Man (3) (17.98%) and complex-type N-glycans such as GlcNAc (4) Man (3) (61.15%). Only a small amount (0.59%) of fucosylated N-glycans was detected (Fig. 3d). From the above results, the rhLF expressed by silkworm has the same amino acid sequence and N-glycosylation modification sites as natural human lactoferrin, but differs in the types of glycosylation modification.

### Inhibitory effects of rhLF on the LPS induced inflammatory response in RAW264.7 macrophage

**Fig. 4:**
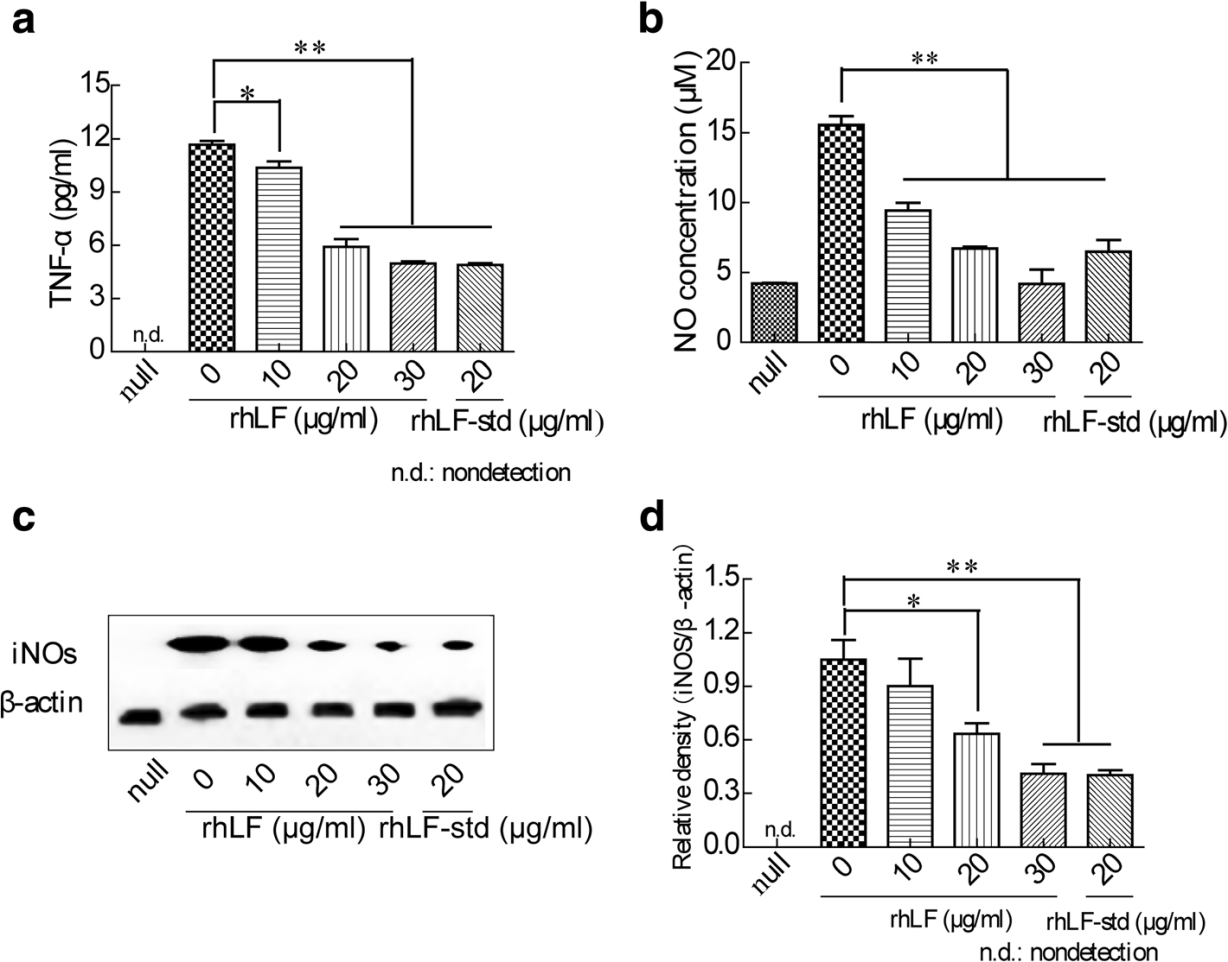
Effect of rhLF on LPS-induced inflammation in RAW264.7 macrophages. Cells at a density of 5 × 10^4^ cells/well were incubated with with a mixture of LPS (200 ng/ml) and various concentrations of purified rhLF for 12 h at 37 °C before the analysis. **a** Accumulated TNF-α in the culture medium was evaluated with a TNF-α detection kit. **b** Accumulated NO in the culture medium was evaluated with a Total Nitric Oxide Assay Kit. **c** The expressions of iNOs and β-actin in cells were determined by western blotting using specific antibodies. **d** Quantification of iNOs expression according to **c** using ImageJ software. The group of null means that rhLF and LPS were not added to the cells. Data shown are the mean ± SD of three experiments and are analyzed by one-way ANOVA, *P < 0.05, **P < 0.01 relative to the control group

LPS-induced macrophage were used as an inflammatory model to test the anti-inflammatory activities of rhLF. LPS successfully induced a dramatic releases of inflammatory factors such as tumor necrosis factor-alpha (TNF-*α*) and nitric oxide (NO), and over expression of the pro-inflammatory nitric oxide synthase (iNOs) in macrophages. The release of TNF-*α* and NO was slightly decreased at the low dose (10 μg/ml) of rhLF treatment. Correspondingly, the release of TNF-*α* and NO was dramatically decreased by 57.50 and 73.05%, respectively, for a high dose (30 μg/ml) of rhLF treatment (Fig. 4a-b), suggesting that rhLF excerts a dosage-dependent inhibition on TNF-*α* and NO release. Expression of iNOs in LPS-induced macrophages was also investigated after rhLF treatment, the results showed that the expression of the pro-inflammatory protein iNOs was decreased with an increasing dose of rhLF treatment (Fig. 4c). These results suggested that rhLF is an effective inhibitor against the LPS-induced inflammatory reaction in RAW264.7 macrophage.

### rhLF inhibits the growths of *Escherichia coli* (*E. coli*) and Bacillus subtilis

**Fig. 5:**
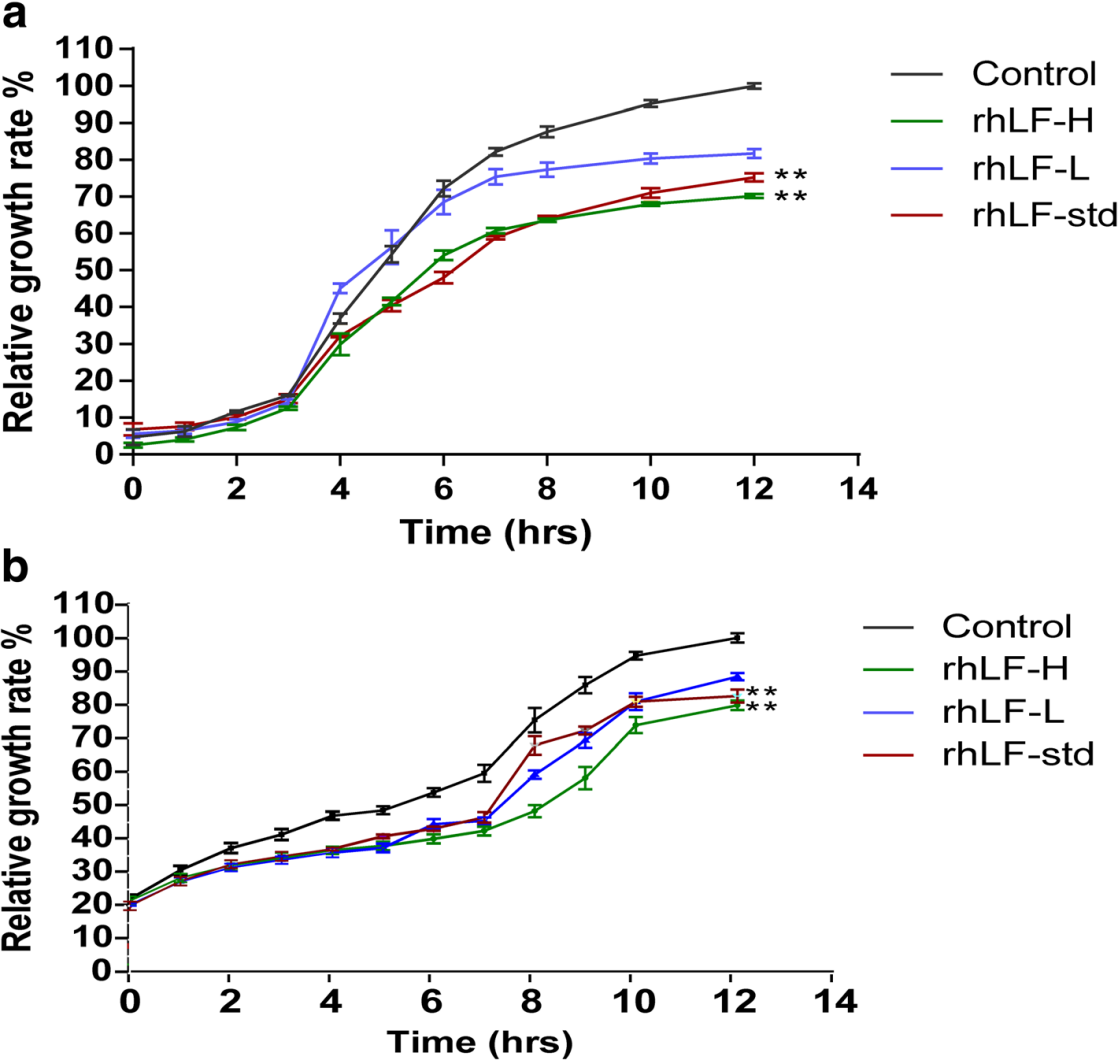
Inhibitory effect of rhLF on the growth of bacteria. **a** Inhibition of rhLF on the growth of *E. coli*. **b** Inhibition of rhLF on the growth of *Bacillus subtilis*. Data shown are the mean ± SD of four experiments and are analyzed by one-way ANOVA, *P < 0.05, **P < 0.01 relative to the control group

**Fig. 6:**
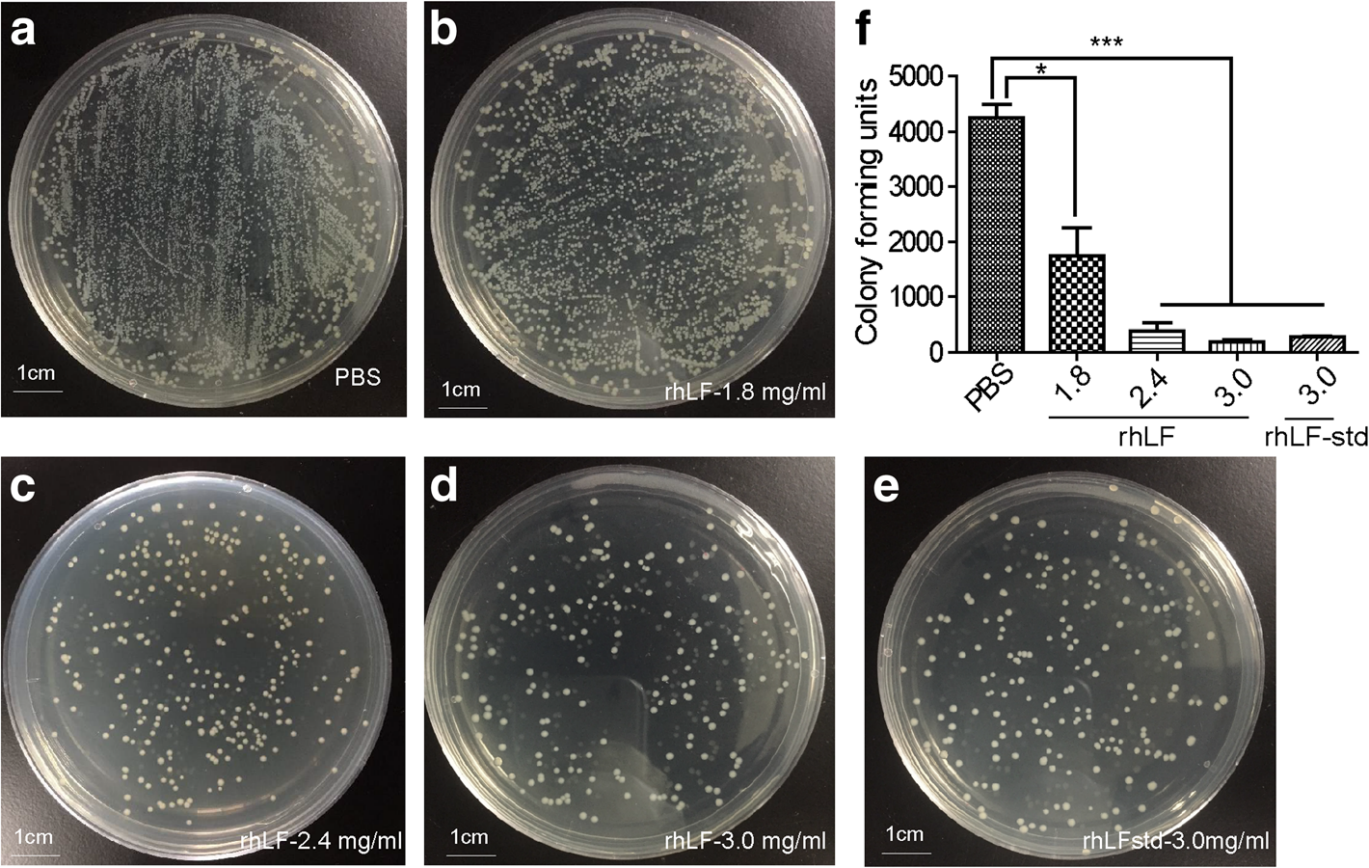
Antibacterial activity analysis of rhLF. (**a**-**e**) *E. coli* was treated with PBS, rhLF, and rhLF-std for 60min, 1 μl of the mixture was plated on LB solid medium and cultured at 37 °C, and then photographed to record colony formation after 18 h. The scale bar is 1 cm. (**f**) The number of *E. coli* colonies was counted according to (**a**-**e**). Data are shown as mean ± SD of three experiments and were analyzed by one-way ANOVA, *P < 0.05, **P < 0.01, ***P < 0.001 relative to the control group

The bacteriostatic effect of rhLF on *E. coli* was investigated. First, several groups of bacteria were each co-incubated with different concentrations of rhLF and their growth was monitored for 12 h. The results showed that the growth of *E. coli* and *Bacillus subtilis* could be significantly inhibited by rhLF with a dose-dependent effect compared to the PBS treatment, which achieved the 20.00 ± 1.11% and 29.85 ± 0.27% suppressions of *E. coli* (Fig. 5a) and 11.57 ± 1.08% and 20.15 ± 1.42% of *Bacillus subtilis* (Fig. 5b) with 0.5 and 2 mg/ml of rhLF, respectively, after the co-incubation for 12 h. In addition, mixtures of *E. coli* with PBS, rhLF, and rhLF-std were inoculated on Luria Bertani (LB) plates and incubated for 18 h; the number of the *E. coli* colonies from each group was then observed and calculated*.* These results showed that *E. coli* mixed with PBS formed the maximum colonies on the LB plate, and that the colonies of *E. coli* mixed with rhLF were significantly reduced in a dosage dependent effect (Fig. 6a-e). The colony count from each group was further calculated and the results showed that when mixed with 1.8, 2.4, and 3.0 mg/ml of rhLF, the colony counts of *E. coli* on the plate reduced to 41.00 ± 11.95%, 8.90 ± 3.54% and 4.48 ± 0.62% of that with the PBS treatment, respectively (Fig. 6f).

## Discussion

Human lactoferrin belongs to the transferrin protein superfamily, which is of considerable interests, and shows a variety of physiological functions, especially involving the non-specific immune system [2, 37, 38]. Considering the potential therapeutic value and the global demand for human lactoferrin, this study attempted to produce the rhLF in the silk gland bioreactor using a transgenic silkworm strategy.

To produce rhLF cost-effectively and at a large scale, we tried to express the rhLF using the previously optimized sericin-1 expression system [29], which resulted in the efficient expression and distribution of rhLF in the sericin layer of the cocoons made using transgenic silkworms. Among the 65 positive silkworm individuals, we found that the expression levels of rhLF varied dramatically, which revealed that the expression of rhLF might suffer because of the chromosome “position effect” [39, 40]. Based on the highest expression and a single copy of transgene insertion principle, individuals with the highest expression were retained and single copy of rhLF was considered a stable transgenic silkworm strain. Production of rhLF by this strategy could be easily scaled up and would be cost-effective, only requiring feeding a mass of silkworms to meet the current pharmaceutical needs and principles.

Interestingly, we found that the expression level of rhLF achieved in this study was higher than that of growth factors [31, 33] produced in the silk glands of transgenic silkworms using a similar expression system. We speculated that the silk gland cells of silkworm likely preferred synthesis of proteins with higher molecular weights and higher abundance, such as human serum albumin [41] and antibodies [32, 39] that exhibit a pattern of high expression levels; these high abundant proteins with various biological activities are crucial for the organism. Further, there is a ubiquitous transferrin with potential immune functions in insects [42] that shows an approximate 30% sequence identity to human lactoferrin, indicating that the human lactoferrin expressed in silk gland cells would not cause serious side effects on the cell metabolism. Thus, it’s reasonable for the silk gland cells of silkworm to adapt the high expression level of proteins with high abundance. These fingding suggest the potential application of silk gland in the recombinant expression of protein with a high molecular weight and high abundance in future research.

The rhLF can be purified conveniently and effectively from cocoons by linking to a 6 × His tag. When the crude extract flows through a nickel ion affinity chromatography column, rhLF binds to the column tightly whereas endogenous sericins do not. Through the repeatable purification process, approximately 9.24 mg of rhLF from 1 g cocoon was purified with a purity of 95% on average. Compared with previous studies, introduction of a 6 × His tag can indeed improve the efficiency of protein purification. However, during the purification process, part of the rhLF was also eluted by low dose imidazole in the elution process, which may be due to the mass molecular weight of rhLF. Thus, further modifying the purification process, such as by enlarging the 6 × His tag to a 12 × His tag may enhance the binding ability of rhLF to the nickel ion affinity chromatography column and improve the purification yield of rhLF.

Glycosylation is a common but complex type of post-translation modification of proteins that directly affects glycoprotein structure, biological function, and recognition [43–45]. Three typical glycosylation modification sites of rhLF were detected. Among them, Asn-138 and Asn-479 have the same modification site as natural human lactoferrin, whereas the third modification site, Asn-624, natural human lactoferrin is usually not modified [46]. In addition, the glycosylation modification type of natural human lactoferrin usually contains a modification type of galactose, whereas the insect expression system does not contain this type of glycosylation modification. In SDS-PAGE analysis (Fig. 3a), the molecular weight of rhLF from silkworm was observed to be different from that of commercial rhLF, probably due to the different glycosylation modifications of the two proteins. Previous studies have shown that both glycosylated and unglycosylated human lactoferrins could bind irons with identical affinity towards human lysozyme and bacterial lipopolysaccharide, but differed in their susceptibilities to tryptic proteolysis [47], and glycosylation modification of lactoferrin protects it from protease hydrolysis. Because the oligomannoside glycans of lactoferrin bind bacterial adhesins, bacterial interaction with host cell receptors is prevented, suggesting that the ability of lactoferrin to prevent the attachment of certain bacteria to the host cell is related to glycosylation modification of lactoferrin [48]. Taken together, glycosylation modification of lactoferrin is very important for the stability and biological activity of lactoferrin. α1, 6-fucosylation may cause immunogenic reactions and commonly exist. In this study, only a small portion of core-fucosylated N-glycans (0.59%) was detected in the rhLF.

Besides, the location of rhLF in the sericin layer of the cocoon confers its convenient and long-term storage. The sericin and fibroin in silk fibers showed the properties of stabilizing collagen, blood components, and antibodies for the long-term stabilities. Thus, the silk fiber can be regarded as a stabilizer for the long-term stability and storage of recombinant proteins produced by the silk gland-based bioreactor. Finally, rhLF could significantly alleviated the LPS-induced inflammation in RAW264.7 cells and inhibit the growth of *E. coli* and *Bacillus subtilis*, which suggested that the rhLF that expressed by the MSG of silkworm also has potential clinical application in disease prevention, treatment and diagnosis.

## Conclusions

In summary, a stable and hereditable transgenic silkworm strain, which produced mass amount of rhLF was first established. The rhLF could be purified with correct protein structure and natural biological activities. These results demonstrated the rhLF recombinant production at large scale and cost effective by feeding transgenic silkworms as alternative potential in the future.

## Materials and method

### Silkworm strain, cells and bacteria

The silkworm, D9L strain was applied to generate transgenic silkworms. Silkworms were reared on fresh mulberry leaves or architecture diets in an artificial climate box at 25 °C temperature, 75% relative humidity, and a photoperiod of 12 h light and 12 h darkness. RAW264.7 cells were cultured in Dulbecco’s modified Eagle medium (DMEM, Gibco, MA, USA) supplemented with 10% fetal bovine serum (FBS, Gibco, MA, USA). *E.coli* and *Bacillus subtilis* were grown in LB-Miller medium.

### Vector construction

Vector construction was performed as per previous reports with some modification [31]. Briefly, the coding sequence of the human lactoferrin gene (GenBank: M93150.1) with 6 × His in the C-terminal was optimized according to the silkworm codon bias and was synthesized commercially. The synthesized human lactoferrin (rhLF) gene was inserted into the previously constructed *sericin-1* transgenic expression vector pSL1180[hr3Ser1DsRedSer1PA] using the *BamH*I and *Not*I restriction endonucleases to replace DsRed, the resulting pSL1180[hr3Ser1rhLFSer1PA] vector was further cut with the *Asc*I restriction endonuclease and the open reading frame (ORF) was then inserted into pBac [3xp3RFPaf] cut using the same enzyme (*Asc*I) to generate the final transgenic expression vector phSrhLFSer1.

### Generation of transgenic silkworm and gene insertion site analysis

Construction of transgenic silkworm was performed according to a previously reported method [25, 49], The rhLF transgenic expression vector phSrhLFSer1 was purified using the QIAGEN Mini kit (Qiagen, Hilden, Germany), mixed with the hsp70-PIG helper [49] vector at a mole ratio of 1:1, and the mixed plasmids with at the concentration of 500 ng/μl were microinjected (Eppendorf, Hamburg, Germany) into non-diapause embryos of the D9L silkworm strain within 2 h after oviposition. The hatched G0 embryos were collected and reared to oviposit G1 eggs. After being developed for 6–7 days, the positive G1 egg individuals were screened for DsRed expression in the eyes using a fluorescence stereomicroscope (Olympus, Tokyo, Japan). The positive G1 individuals were hatched and reared with wild type larva to the moth stage, and were backcrossed with wild type moths to generate stable transgenic silkworms. The cocoons from the positive individuals were collected for protein expression analysis.

### Expression analysis of rhLF from transgenic silkworm

Cocoons from the G1 positive individuals were independently frozen in liquid nitrogen, powdered, and then added to the extraction solution containing 8 M urea, 50 mM Tris-HCl, pH 8.0 at a final concentration of 20 mg/ml for 40 min under 80 °C. The supernatant was collected by centrifugation at 20,000×g for 10 min. Next, 40 μl supernatant proteins together with different concentration (100, 250, 500 mg/mL) of commercially purchased lactoferrin standard (Sigma-Aldrich, Saint Louis, USA) were mixed with 10 μl proteins loading buffer and denatured for 5 min at 95 °C. The samples were analyzd on 4–20% SDS-PAGE (sodium dodecyl sulfate-polyacrylamide gel electrophoresis) gels (Genscript, Nanjing, China) analyzed, and stained with Coomassion brilliant blue (CBB) R-250. A gel of a parallel set was further electronically transferred to the polyvinylidene fluoride (PVDF) membrane, followed by the immunoreaction with a rabbit anti-human lactoferrin antibody (Sigma-Aldrich, Saint Louis, USA) at a 1:1000 dilution, 25 °C, for 1 h and was further incubated with anti-rabbit IgG conjugated with horseradish peroxidase (Beyotime, Shanghai, China) as the secondary antibody at 25 °C for 1 h, and the protein was finally detected with an enhanced chemiluminescence (ECL) western blotting kit (Thermo Fisher, USA). Images were immediately recorded on a Chemiscope Series (Clinx Science Instruments, Shanghai, China). The content of lactoferrin in the cocoon shell by weight was quantified through densitometric analysis of the intensity of the extracted lactoferrin with rhLF-std on the western blots using Image J software (http://rsb.info.nih.gov/ij/). The positive moth corresponding to the cocoon with the highest expression of lactoferrin was collected for further genome analysis and its oviposition was regarded as the stable transgenic train for further use.

### Inverse PCR to analysis of the genome integration of transgene

Genomic DNA from the positive moth with the highest expression of lactoferrin was extracted for inverse PCR analysis. Briefly, 20 μg genomic DNA was digested with *Hae*III at 37 °C overnight and was purified using the phenol/chloroform method. Then, 2 μg was circularized by ligation using T4 DNA ligase at 16 °C overnight. The ligated DNA (50–100 ng) was amplified using the primers: Transposon-specific pBacF: 5′-TACGCATGATTATCTTTAACGTA-3′ and pBacR: 5′-GTACTGTCATCTGATGTACCAGG-3′. Amplified products were extracted with an OMEGA Gel Extraction Kit (Omega Bio-Tek, Guangzhou, China), and then inserted into the TA-cloning vector (TAKARA Bio, Dalian, China) for sequencing and analysis according to the silkworm genome database: SilkDB (http://www.silkdb.org/silkdb/).

### Purification of the rhLF from the cocoons

Cocoons from the silkworm strain with the highest expression of lactoferrin were frozen in liquid nitrogen and shattered into powder. The crude cocoon proteins were extracted and collected. Subsequently, the crude extract was slowly past HisTRap™ FF (GE Healthcare, Uppsala, Sweden) column. Elution buffers with different concentrations of imidazole (0, 20, 35, 50, 100, 200 mM) were used for gradient elution. Flowing samples were collected for SDS-PAGE and western blotting analysis to determine the wash and elution, respectively. Then, the eluted rhLF proteins with high purity were collected and dialyzed (cellulose dialysis membranes, MWCO 14,000 Da, Sangon, China) against the dialysis solution (50 mM Tris–HCl, pH 7.0, 250 mM NaCl) for 3 days at 4 °C. The dialysis solution was replaced every 12 h to completely remove the urea and imidazole. The purified rhLF was then analyzed by 4–20% SDS-PAGE. The concentration of the crude extract and purified rhLF was calculated by densitometric measurements of the protein bands compared with the rhLF-std (Sigma-Aldrich, Saint Louis, USA) on the immunoblot using Image J software (http://rsb.info.nih.gov/ij/download.html).

### Secondary structure analysis of the purified rhLF

The purified rhLF at a concentration of 0.5 mg/ml was used for secondary structure analysis by obtaining far-UV CD spectra using a 0.05 cm quartz cell at 25 °C and 190–250 nm using standard procedures. The rhLF-std with the same concentration was used as the positive control.

### Analysis of the rhLF N-glycosylated modification

N-glycosylation modification of rhLF was determined according to a previous study [50], with some changes. Briefly, the purified rhLF was determined by SDS-PAGE and stained with CBB, the specific band was recovered in a 1.5-ml EP tube. Next, 30% acetonitrile (ACN)/H_2_O was added to the tube and shaken until the gel pieces became clear. Destaining buffer was removed, 10 mM dithiothreitol (DTT) and 50 mM Miodoacetamide (IAA) were used for breaking and blocking the disulfide bonds, respectively. All the buffer was then discarded, the gels were cleaned with 100 μl of 100 mM NH_4_HCO_3_, and then lyophilized. The gels were digested using 5 μl of 10 ng/μl Trypsin in 50 mM NH_4_HCO_3_ at 4 °C for 30 mins, then added an appropriate volume of 50 mM NH_4_HCO_3_ to the tube to ensure that all the gels were covered with digestion buffer, followed by incubation at 37 °C for 16 h. Digestion buffer was collected, 100 μl of 60% ACN/0.1% TFA was added to the gels and sonicated for 15 mins. All the buffer was collected and mixed with the digestion buffer. Peptides were lyophilized and stored at − 80 °C before use. A raw file was acquired with data dependent acquisition mode using Orbitrap Fusion Lumos (San Jose, Thermo Fisher). A home-made C18 column (3 μm, 75 μm × 15 cm) at a flowrate of 600 nl/min was used to separate the peptide mixture on an EasyNano LC1000 system (San Jose, Thermo Fisher) using a home-made C18 column (3 μm, 75 μm X 15 cm) at a flowrate of 600 nl/min. A 60-min linear gradient was set as follows: 3% B (0.1% FA in ACN)/ 97% A (0.1% FA in H2O) to 8% B in 5 mins, 8% B to 20% B in 38 mins, 20% B to 30% B in 8 mins, 30% B to 90% B in 2 mins and hold for 7 mins at 90% B. For the data acquisition a top 20 scan mode with MS1 scan range m/z 350–1550 was used and other parameters were set as below: MS1 and MS2 resolution was set to 120 K and 30 K; AGC for MS1 and MS2 was 4e5 and 1e5; isolation window was 1.6 Th, dynamic exclusion time was 15 s. The collision method of EThcD was applied to each parent ion in order to identify the glycosylated amino acid sites. HCD energy was set to 25, and ETD reaction time was set automatically according to m/z and ion charge state of each precursor.

Data Analysis: Raw file against the target protein sequence was searched using Byonic v2.16.11 (Protein Metrics). Search parameters were set as follows: enzyme of trypsin (semi) with maximum number of 2 missed cleavages; precursor and fragment ion mass tolerance was set to 10 ppm and 0.02 Da, respectively; variable modification was set to oxidation of M and deamidation of N, Q and fixed modification was set to carbamido methylation of C; a 182 kinds of N-glycan modification in the database were also searched against and an automatic score cut was used to remove low confidence peptides. A much stricter rule was used as below: Score ≥ 300, |Log Prob| ≥ 3, Delta Mod≥10 for further filtering high confidence glycosylated peptides. Byologic (Protein Metrics) was used to further process the result. The glycosylated ratio of each potential site was quantitated using the peptide’s XIC-AUC (Extracted Ion Current-Area Under Curve).

### Anti-inflammatory activities of rhLF

RAW264.7 macrophages were added into a 96-well plate at a density of 5 × 10^4^ cells/well and grown for 12 h in DMEM medium which containing 10% FBS. Subsequently, the culture medium was discarded, replenished with a mixture of LPS (200 ng/ml) and various concentrations of purified rhLF (10, 20, 30 μg/ml), and then incubated for another 12 h. After incubating, the cell culture medium was collected, and used for evaluating NO and TNF-α using the by Total Nitric Oxide Assay Kit (Beyotime, Shanghai, China) and TNF-α detection kit (R&D, Minnesota, USA). Further, the cells were washed twice with phosphate buffer solution and lysed with RIPA lysate (Beyotime, Shanghai, China); the lysed cells were used to detect the expression of iNOS expression by immunoblotting, the primary antibodies used were the anti-iNOS antibody and anti-β-actin antibody (abcam, Cambridge, UK), and the secondary antibodies were the rabbit IgG antibody and mouse IgG antibody (Beyotime, Shanghai, China). PBS and LPS were added as blank control and rhLF-std was used as the positive control in this experiment, with three replicates per group.

### Antibacterial activity of rhLF

*E.coli*, DH5a and *Bacillus subtilis* were activated by overnight cultures; 200 μl *E.coli* and *Bacillus subtilis* cultures were inoculated into a 10 ml tube containing 5 ml LB, and incubated the tube at 37 °C overnight with shaking (200 rpm). Next, 200 μl the overnight *E. coli* and *Bacillus subtilis* cultures were added to 5 ml of fresh LB medium and incubated for another 1 h. Then, 100 μl of bacteria culture was added to a 96-well plate and incubated with 100 μl of purified rhLF at various concentrations (0.5 and 2 mg/ml), PBS as the blank control, and 2 mg/ml rhLF-std as the positive control. Bacterial growth was measured using a Microplate Reader (OD_600nm_) at every 1 h for 12 h. Four repetitions were performed each group.

Next, 50 μl of activated bacterial cultures were mixed with 50 μl PBS, rhLF-std (3.0 mg/ml), and various concentrations (1.8, 2.4, 3.0 mg/ml) of purified rhLF, and incubated at 37 °C for 60 min with shaking (200 rpm); then 1 μl of the mixture was plated on LB solid medium and observed after 18 h. Three repetitions were performed per group.

### Statistics

Data are presented as mean ± SD (*n* = 3 or 4). Statistical analyses were performed using performed using SPSS 20.0 software (SPSS Inc., Chicago, IL, USA). *P* <0.05 was considered statistically significant.

## Additional files

Additional file 1: **Figure S1.** Screening of transgenic silkworm eggs, pupae and moth. (a) Fluorescence image of a transgenic silkworm egg. (b) Fluorescence image of a transgenic silkworm pupa. (c) Fluorescence image of a transgenic silkworm moth. (d) White light image of a transgenic silkworm egg. (e) White light image of a transgenic silkworm pupa. (f) White light image of a transgenic silkworm moth. (TIF 6774 kb)

Additional file 2: **Figure S2.** SDS-PAGE analysis of rhLF in cocoon from 65 positive silkworm individuals. M and WT represent the marker and wild type cocoons, respectively. (TIF 2197 kb)

Additional file 3: **Figure S3.** SDS-PAGE analysis of rhLF in the purification process using a Ni-charged His-binding column. M and WT represent the marker and wild type cocoons, respectively. Lane 1 represents crude extract from rhLF cocoons. Lane 2 represents the constituents flowing through the column. Lane 3–7 represent the rhLF with elution buffers containing 20 mM, 35 mM, 50 mM, 100 mM, and 200 mM imidazole, respectively. (TIF 2709 kb)

Additional file 4: **Figure S4.** Inverse PCR-amplified products of rhLF from the genomic DNA. M represents the marker; Lane 1 represents PCR-amplified products. (TIF 994 kb)

Additional file 5: **Figure S5.** Amino acid sequence matching map of rhLF. Red labeled amino acid sequences were identified, whereas none were black labeled. (TIF 2785 kb)

## Abbreviations

CBB: Coomassion brilliant blue
CD: Circular dichroism
DMEM: Dulbecco’s modified eagle medium
*E. coli*: *Escherichia coli*
FBS: Fetal bovine serum
iNOs: nitric oxide synthase
LB: Luria Bertani broth
LC/MS: Liquid chromatography/mass spectrometry
LPS: Lipopolysaccharide
MSG: Middle silk gland
NO: Nitric oxide
ORF: Open reading frame
PVDF: Polyvinylidene fluoride
rhLF: recombinant human lactoferrin
rhLF-std: recombinant human lactoferrin standard
SDS-PAGE: Sodium dodecyl sulfate-polyacrylamide gel electrophoresis
TNF-α: Tumor necrosis factor-alpha
