# Supplementary figures and images for: A silkworm based silk gland bioreactor for high-efficiency production of recombinant human lactoferrin with antibacterial and anti-inflammatory activities
Source: J Biol Eng. 2019 Jul 5;13:61. doi: 10.1186/s13036-019-0186-z (PMC6612213; doi:10.1186/s13036-019-0186-z)

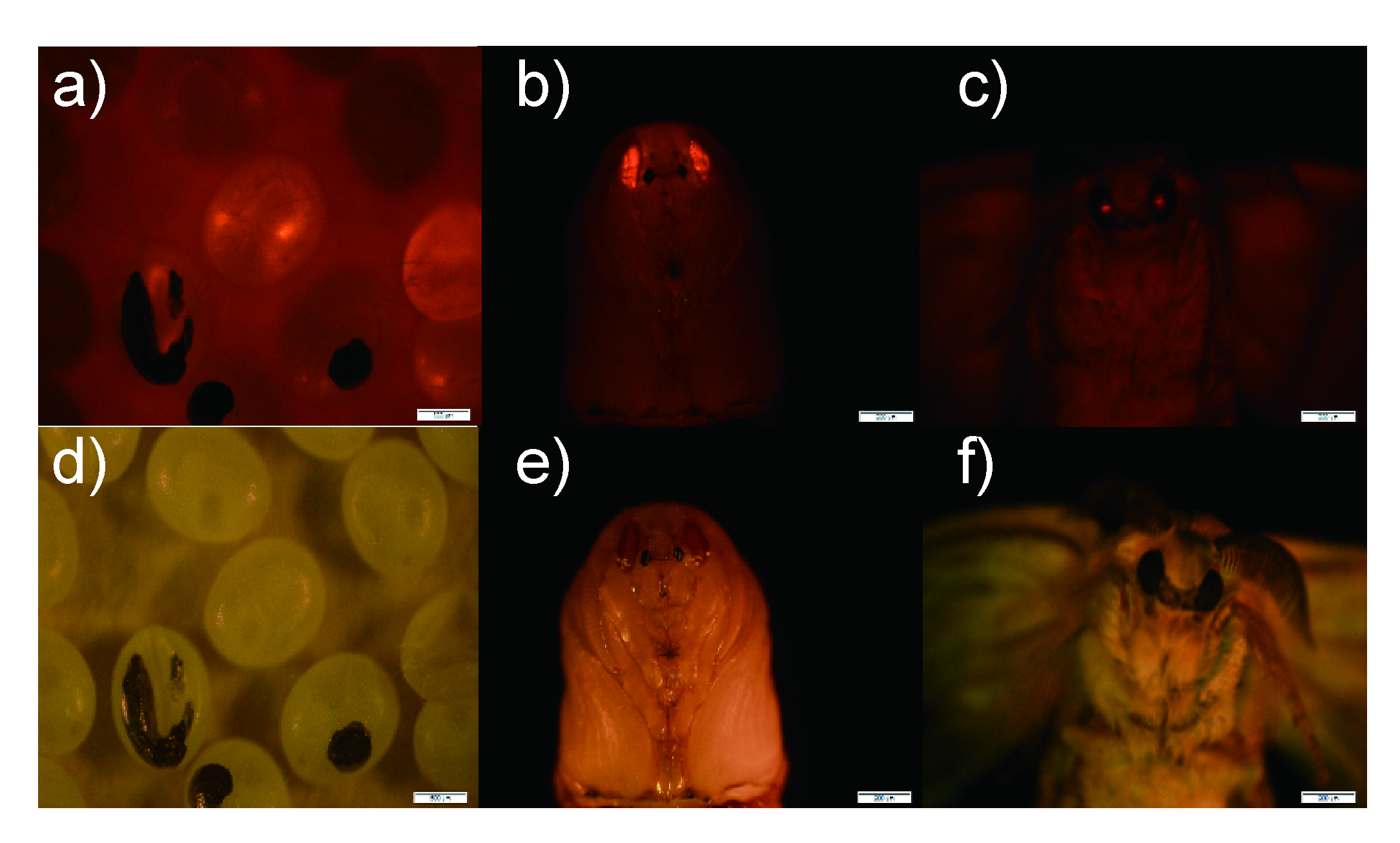

Supplement: Supplementary file 1 — Figure S1. Screening of transgenic silkworm eggs, pupae and moth. (a) Fluorescence image of a transgenic silkworm egg. (b) Fluorescence image of a transgenic silkworm pupa. (c) Fluorescence image of a transgenic silkworm moth. (d) White light image of a transgenic silkworm egg. (e) White light image of a transgenic silkworm pupa. (f) White light image of a transgenic silkworm moth. (TIF 6774 kb) [file 13036_2019_186_MOESM1_ESM.tif]

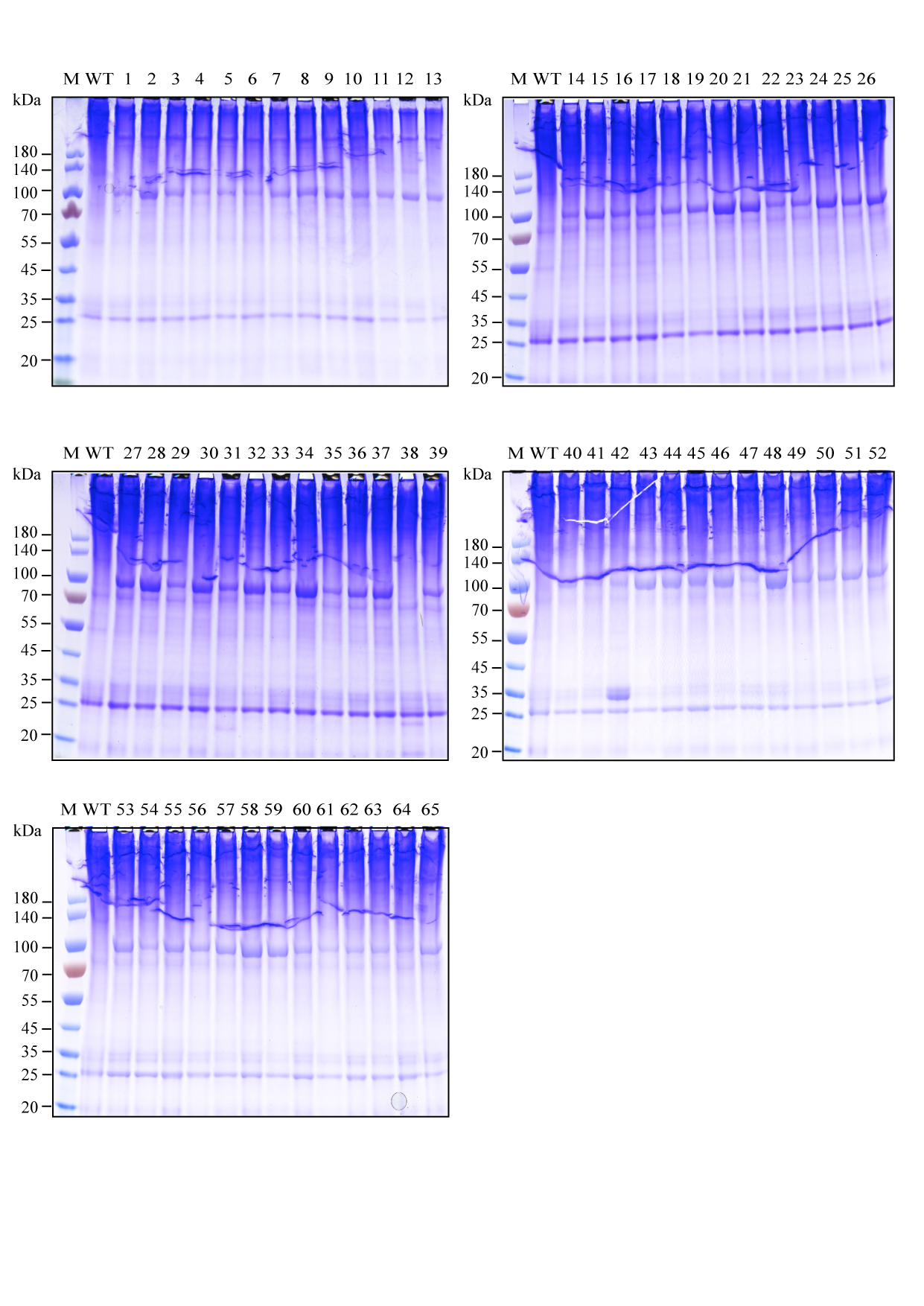

Supplement: Supplementary file 2 — Figure S2. SDS-PAGE analysis of rhLF in cocoon from 65 positive silkworm individuals. M and WT represent the marker and wild type cocoons, respectively. (TIF 2197 kb) [file 13036_2019_186_MOESM2_ESM.tif]

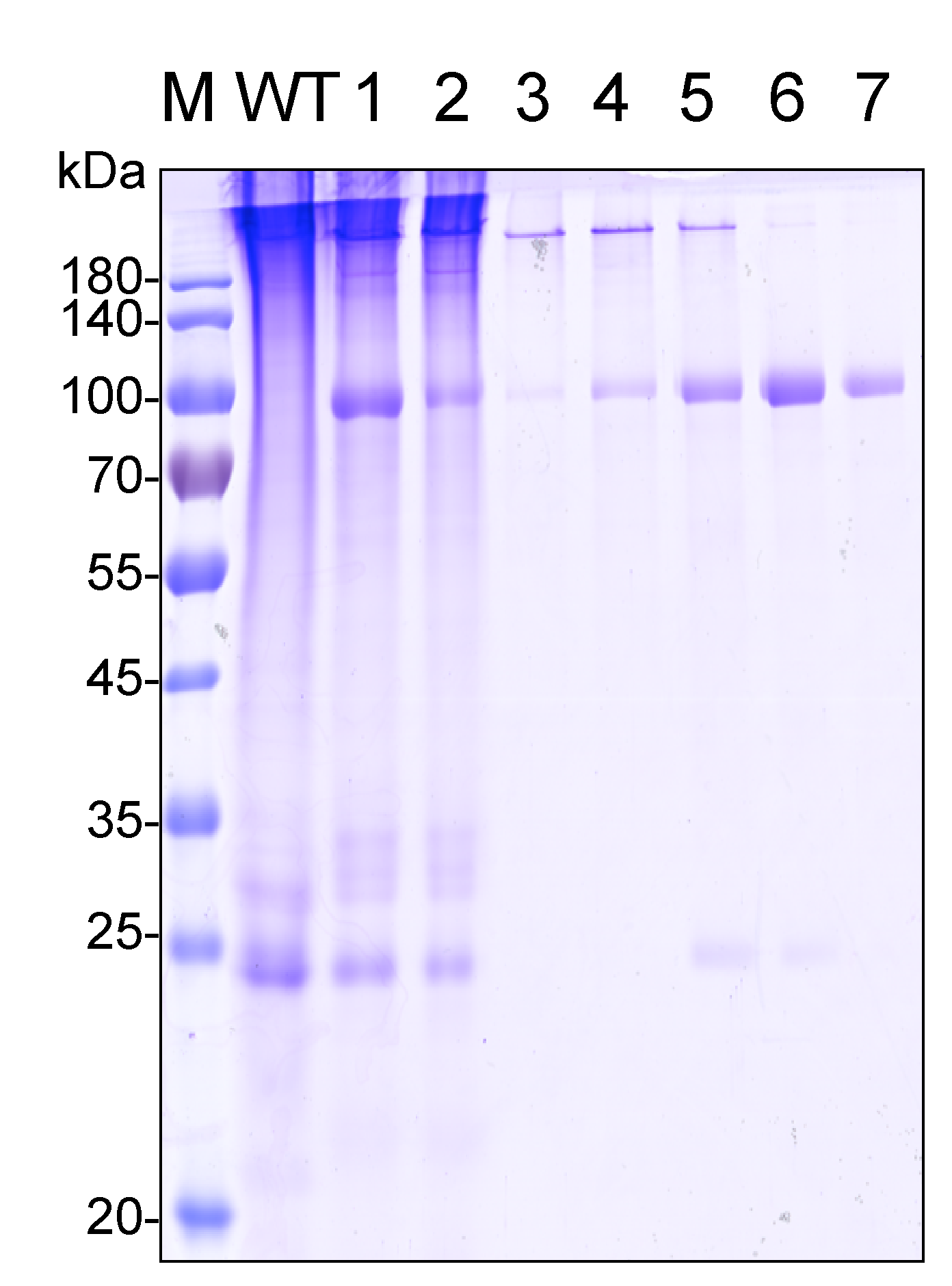

Supplement: Supplementary file 3 — Figure S3. SDS-PAGE analysis of rhLF in the purification process using a Ni-charged His-binding column. M and WT represent the marker and wild type cocoons, respectively. Lane 1 represents crude extract from rhLF cocoons. Lane 2 represents the constituents flowing through the column. Lane 3–7 represent the rhLF with elution buffers containing 20 mM, 35 mM, 50 mM, 100 mM, and 200 mM imidazole, respectively. (TIF 2709 kb) [file 13036_2019_186_MOESM3_ESM.tif]

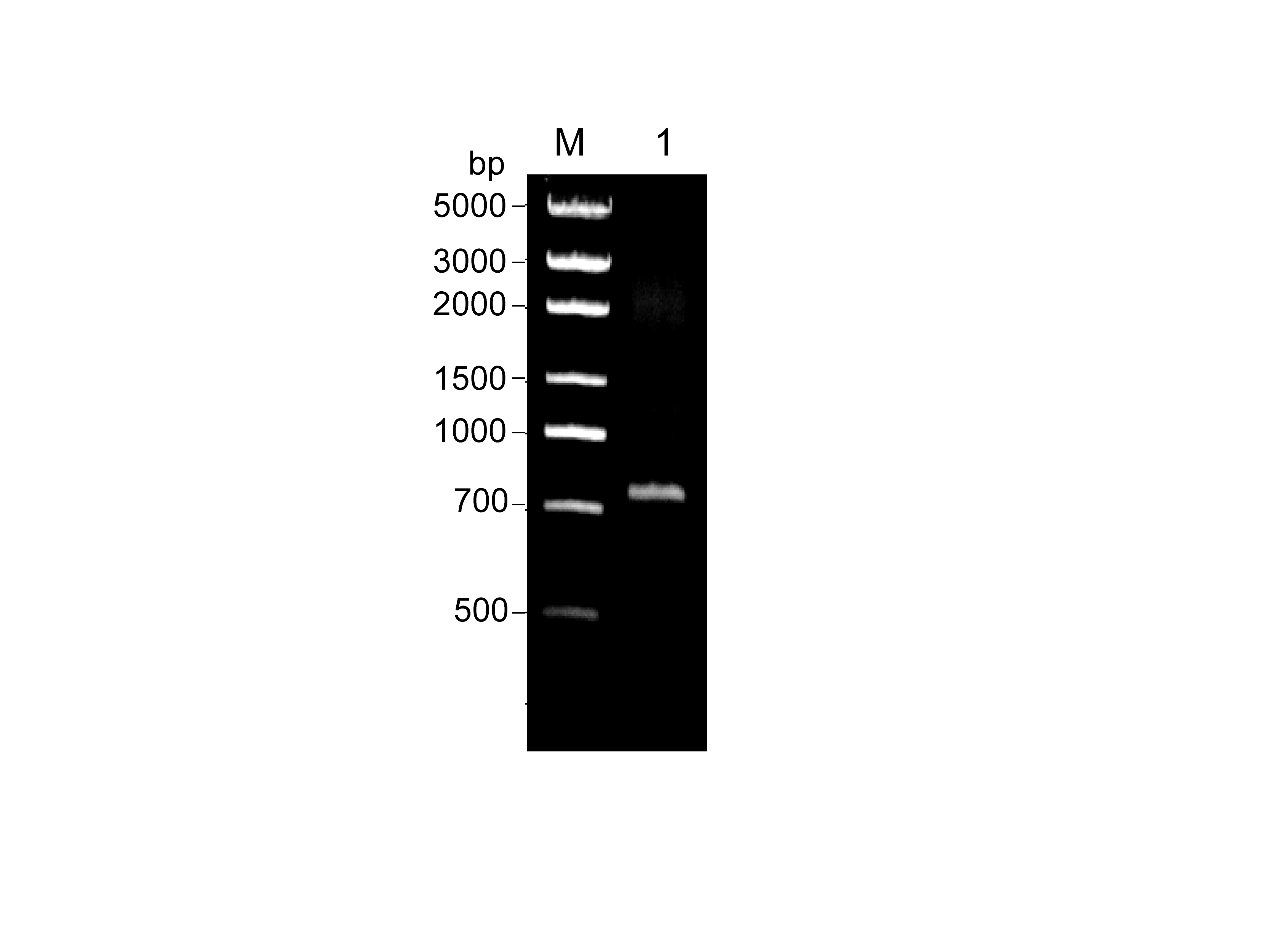

Supplement: Supplementary file 4 — Figure S4. Inverse PCR-amplified products of rhLF from the genomic DNA. M represents the marker; Lane 1 represents PCR-amplified products. (TIF 994 kb) [file 13036_2019_186_MOESM4_ESM.tif]

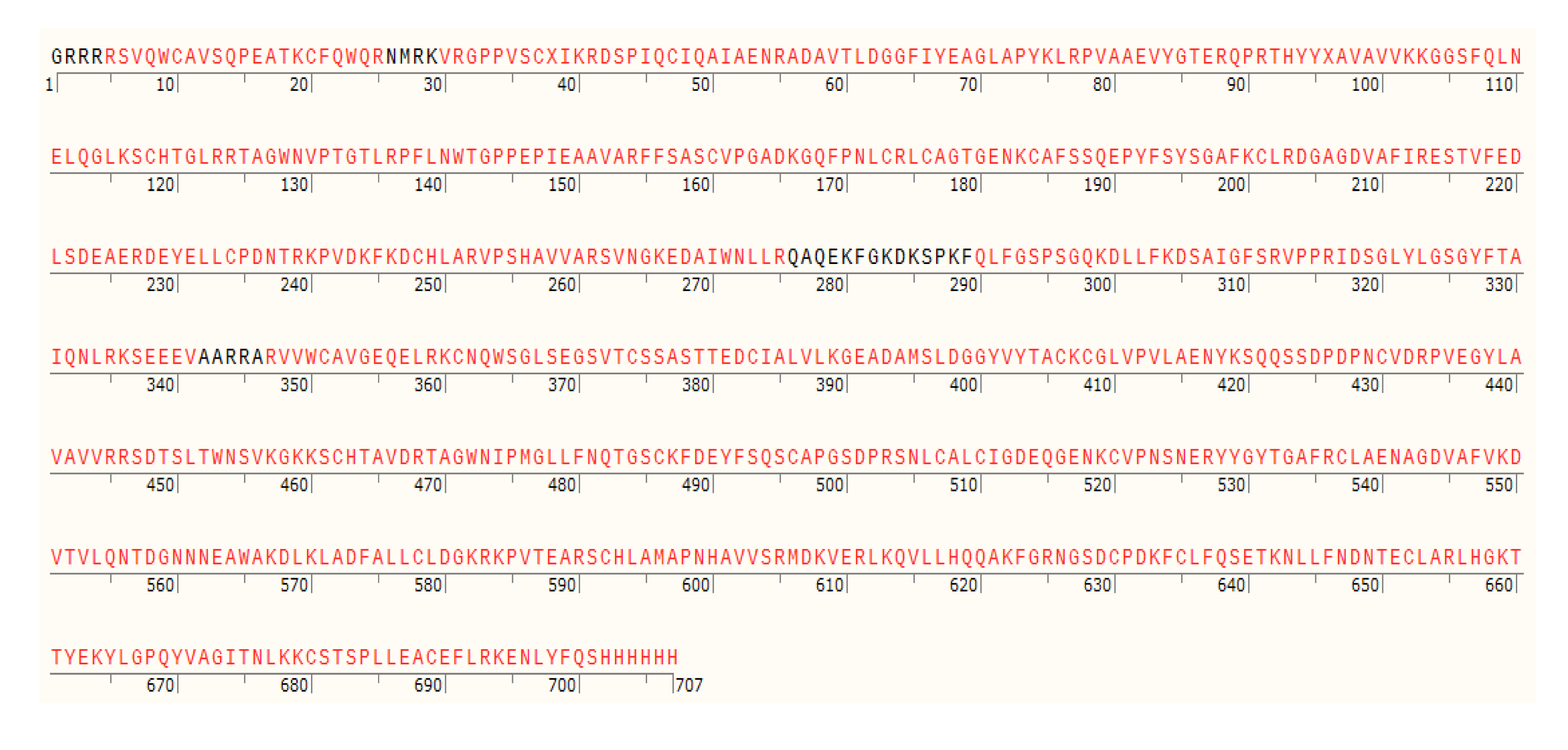

Supplement: Supplementary file 5 — Figure S5. Amino acid sequence matching map of rhLF. Red labeled amino acid sequences were identified, whereas none were black labeled. (TIF 2785 kb) [file 13036_2019_186_MOESM5_ESM.tif]
